# Can routine perioperative haemodynamic parameters predict postoperative morbidity after major surgery?

**DOI:** 10.1186/s13741-020-0139-6

**Published:** 2020-03-24

**Authors:** Jean-Francois Bonnet, Eleanor Buggy, Barbara Cusack, Aislinn Sherwin, Tom Wall, Maria Fitzgibbon, Donal J. Buggy

**Affiliations:** 1 Department of Anaesthesiology & Perioperative Medicine, Mater University Hospital, School of Medicine, University College Dublin, Dublin, Ireland; 2 Department of Medical Biochemistry, Mater University Hospital, School of Medicine, University College Dublin, Dublin, Ireland

**Keywords:** Haemodynamic parameters, Predictor, Morbidity, Intraoperative, Complications, NT-ProBNP, Pulse pressure, Hypotension

## Abstract

**Background:**

Postoperative morbidity occurs in 10–15% of patients undergoing major noncardiac surgery. Predicting patients at higher risk of morbidity may help to optimize perioperative prevention. Preoperative haemodynamic parameters, systolic arterial pressure (SAP) < 100 mmHg, pulse pressure (PP) > 62 mmHg or < 53 mmHg, and heart rate (HR) > 87 min^-1^ are associated with increased postoperative morbidity. We evaluated the correlation between these and other routine haemodynamic parameters, measured intraoperatively, with postoperative morbidity. Postoperative morbidity was measured using the Comprehensive Complication Index (CCI) and length of stay (LOS). Additionally we correlated CCI with the cardiac risk biomarker, preoperative NT-ProBNP.

**Methods:**

This is a retrospective analysis of patients in MET-REPAIR, a European observational study correlating self-reported physical activity with postoperative morbidity. Patients’ electronic anaesthetic records (EARs) including perioperative haemodynamic data were correlated with 30-day postoperative morbidity, CCI and LOS parameters. Statistical analysis to assess for correlation was by Kendall’s Correlation Coefficient for tied ranks (Tau-B) or Spearman’s Correlation Coefficient. Blood for N-terminal prohormone of brain natriuretic peptide (NT-proBNP) measurement was collected < 31 days before surgery.

**Results:**

Data from *n* = 50 patients were analysed. When stratified according to age > 70 years and ASA > 3, the duration of MAP < 100 mmHg, < 75 mmHg or < 55 mmHg were associated with a higher CCI (tau = 0.57, *p* = 0.001) and duration < 75 mmHg was associated with prolonged LOS (tau = 0.39, *p* = 0.02). The intraoperative duration of PP > 62 mmHg was associated with LOS (tau = 0.317, *p* = 0.007). There was no correlation between preoperative NT-proBNP and either CCI or LOS.

**Conclusions:**

In older and higher risk patients, duration of intraoperative hypotension by a variety of definitions, or PP > 62 mmHg, are associated with increased postoperative CCI and LOS. These findings warrant confirmation in larger databases with evaluation of whether real-time intraoperative intervention could reduce postoperative morbidity.

## Introduction

More than 300 million surgical procedures are undertaken globally per annum (Rose et al. 2015). Up to one-third of these patients are at increased risk of cardiovascular or other postoperative morbidity and mortality (Kristensen et al. 2014a). Indeed 10–15% of patients undergoing major noncardiac surgery, who have at least one identifiable preoperative risk factor, develop a significant postoperative complication (Cramer et al. 2014; Wesselink et al. 2018). Predicting which patients are at greatest risk of postoperative adverse events is important to enable perioperative care to be focussed on prevention and early intervention.

Large observational studies have previously shown an association between a number of routine preoperative haemodynamic parameters such as duration of pulse pressure (PP) > 62 mmHg or < 53 mmHg, systolic arterial pressure (SAP) < 100 mmHg or > 160 mmHg, heart rate (HR) < 55 or > 100 min^-1^ and myocardial injury after noncardiac surgery (MINS), acute kidney injury (AKI) or death (Abbott et al. 2017a; Abbott et al. 2017b; Ackland et al. 2018; Botto et al. 2014).

Baseline preoperative haemodynamic parameters are challenging to standardise, and debate continues as to whether benchmark values should be taken from the preoperative assessment clinic or ward data, or immediately prior to induction of general anaesthesia. Multidisciplinary guidelines on intraoperative anaesthetic haemodynamic management highlight the absence of universally agreed intraoperative target blood pressure values (Sessler et al. 2019).

The advent of electronic anaesthetic records (EARs) increasingly enables accurate, real-time documentation of variations and trends in these haemodynamic parameters in the intraoperative period during anaesthesia. Whether these intraoperative haemodynamic parameters during anaesthesia and surgery correlate with cumulative postoperative morbidity has not been evaluated. Therefore, among a study group of increased risk patients undergoing major noncardiac surgery, we correlated haemodynamic parameters measured electronically during anaesthesia with postoperative morbidity, measured by the Comprehensive Complications Index (CCI) (de la Plaza Llamas et al. 2018) and length of hospital stay (LOS).

## Methods

In our hospital, we routinely record intraoperative haemodynamic parameters in an electronic anaesthesia record (EAR). We took a subset of patients (*n* = 50) recruited for the European MET-REPAIR study, and retrospectively observed the correlation between intraoperative haemodynamic parameters and postoperative morbidity.

MET-REPAIR is a prospective observational study of > 15,000 patients, organised by the European Society of Anaesthesiology Clinical Trials Network. The study objective is to correlate patients’ self-reported activity levels, described in terms of number of metabolic equivalents (METS) with 30-day postoperative morbidity, among increased risk patients undergoing major noncardiac surgery.

Accordingly, as an enrolling centre for MET-REPAIR, we had a cohort of increased risk patients undergoing major noncardiac surgery, whose 30-day postoperative morbidity had been tracked. While preoperative and some intraoperative data have previously been shown to be correlated with these adverse outcomes (Abbott et al. 2017a; Abbott et al. 2017b; Ackland et al. 2018; Botto et al. 2014; Cramer et al. 2014; Kristensen et al. 2014b; Wesselink et al. 2018), intraoperative haemodynamic parameters, recorded digitally during anaesthesia on our EAR, have never been scrutinised for their predictive potential for cumulative postoperative morbidity. Therefore, we evaluated the correlation between intraoperative haemodynamic parameters and postoperative morbidity, measured by an incremental scale called the Comprehensive Complications Index (CCI) (de la Plaza Llamas et al. 2018).

Institutional Review Board (IRB) approval was granted for MET-REPAIR. Separately, IRB approval was granted for the present study, with a waiver of consent, because these patients had already consented to their perioperative clinical course and data being tracked and documented. The distinguishing feature of this study is the focus on their data recorded during anaesthesia. Sample size calculation was made based on convenience, meaning all of the available patients recruited between January 2019 and June 2019 who had completed their surgery and 30-day postoperative observation period were included for analysis.

Inclusion and exclusion criteria reflected that of the MET-REPAIR study. Inclusion criteria were that patients were inpatients, > 45 years, undergoing elevated-risk elective noncardiac surgery. This was defined as either Revised Cardiac Risk Index (RCRI) > 2 or a National Surgical Quality Improvement Program (NSQIP) > 1%. The NSQIP is in turn defined as the risk (%) of perioperative cardiac events using the American College of Surgeons (ACS) ACS-NSQIP prediction tool that was being used at the time of the MET-REPAIR study. Patients were also included if aged > 65 years and undergoing intermediate or high-risk procedures regardless of their RCRI or NSQIP scores according to ESA guidelines, and having given written informed consent.

Exclusion criteria were patients having emergency surgery, acute coronary surgery or uncontrolled congestive heart failure within the last 30 days, stroke within the last 7 days of planned day of surgery, and patients unable to ambulate due to congenital or chronic illnesses.

The study was limited to the review of existing medical records of patients enrolled in MET-REPAIR in our centre. Data sources used included paper medical records, pre-anaesthetic assessment forms, intraoperative EAR and results of preoperative serum of N-terminal prohormone of brain natriuretic peptide (NT-proBNP) samples.

As part of the MET-REPAIR study, we had previously obtained data on patients’ preoperative maximum MET value from the Adult Compendium of Physical Activities. The validated Rockwood Clinical Frailty scale was used to assign patient-specific numbers based on the level of preoperative independence in the activities of daily living (ADL) and amount of weekly physical activity. This information was obtained retrospectively from patient-reported functional activities in the MET-REPAIR clinical research form section about the level of independence. The CCI is based on the complication grading using the Clavien-Dindo Classification and takes every complication event after surgery into account. An overall patient morbidity score is reflected on a scale, with higher values indicating more severe postoperative morbidity (de la Plaza Llamas et al. 2018). The patient's medical records and data analysed for MET-REPAIR were used to populate this calculator. LOS was also calculated for each patient.

### Statistical methods

Data analysis was performed using SPSS version 16 (IBM, NY, USA) and analysed for distribution. Descriptive statistics were calculated initially and expressed as mean (SD), median (25%–75% range) or number (%), as appropriate.

As much of the data were nonparametric, correlation between patient characteristics, intraoperative haemodynamics and postoperative CCI and length of stay (LOS) was conducted using the correlation coefficient for tied ranks (Tau-B), or Spearman’s correlation coefficient for NT-proBNP data. Values of *p* < 0.05 were deemed statistically significant.

## Results

Data from n = 50 patients were analysed, with baseline characteristics summarised in Table 1. The most frequent indication for surgery was treatment for active cancer, while hypertension, high BMI and COPD were among the most common comorbidities observed. Baseline haemodynamic parameters shown are the mean values just prior to induction of anaesthesia, recorded in the preoperative anaesthesiology induction room (Table 2).

**Table 1:** Patient characteristics

Age (year)	70.1 (7.0)
Gender (M, F)	16, 34
Surgery type:	
Intra-abdominal	30 (60)
Thoracic	12 (24)
Other	8 (16)
Length of stay in PACU (min)	135 (45)
Length of stay in hospital (days)	7.2 (3–10)
Length of stay in ICU (days)	1.1 (0–2)
ASA Grade	
II	15 (30)
III or IV	35 (70)
BMI	27.9 (5.5)
Frailty scale	2 (2–3)
Metabolic equivalents (MET)	4 (2–6)
Diabetes	8 (16)
Hypertension	35 (70)
Congestive cardiac failure	3 (6)
Coronary artery disease	11 (22)
Previous MI	2 (4)
Percutaneous coronary intervention (PCI)	5 (10)
Peripheral vascular disease	10 (20)
Surgery for cancer this admission	35 (70)
Chronic obstructive pulmonary disease	14 (28)
Preoperative creatinine (μmol L^-1^)	82 (21)
Postoperative creatinine (μmol L^-1^)	84 (32)
NSQIP (%)	14.4 (12.7–16.0)
Duration of surgery (min)	180 (103)
Anaesthetic technique:	
GA alone	24 (48)
Regional alone	8 (16)
Combined	18 (36)

All data are shown as mean (SD), median (25%—75% range) or *N* (%)

**Table 2:** Preoperative and intraoperative haemodynamic parameters

Baseline HR (min^-1^)	69 (33)
Baseline SAP (mmHg)	155 (35)
Baseline MAP (mmHg)	97 (25)
Baseline pulse pressure (mmHg)	66 (38)
Highest intraoperative HR (min^-1^)	93 (58)
Duration HR > 100 (min)	51 (40)
Duration HR < 55 (min)	1 (0–1)
Duration HR > 87 (min)	0 (0–8)
Duration SAP < 100 (mmHg)	43 (19–71)
Duration pulse pressure > 62 (min)	10 (3–38)
Duration pulse pressure < 53 (min)	71 (32–115)
Lowest MAP (mmHg)	54 (10)
Duration MAP < 75 (min)	66 (33–120)
Duration MAP < 55 (min)	0 (0–3)

All data shown are mean (SD)

A list of univariate correlations with postoperative morbidity, using the CCI and LOS are shown in Table 3. Duration (minutes) of MAP < 75 mmHg was associated with increased CCI (tau = 0.319, *p* = 0.03) while duration of PP > 62 mmHg was correlated with LOS only (tau = 0.317, *p* = 0.03). There was no correlation between any haemodynamic parameter and duration of stay in the Post Anaesthesiology Care Unit (PACU). None of the Kendall’s Correlation Coefficient for tied ranks (Tau-B) values in the PACU reached statistical significance.

**Table 3:** Correlations between patient characteristics, intraoperative haemodynamic parameters and Comprehensive Complication Index and length of stay

	Comprehensive Complications Index		Length of hospital stay	
	Tau	*p* value	Tau	*p* value
General				
Comprehensive Complication Index	1			
Length of stay in hospital (total)	0.374*	0.005	1	
Age	0.286*	0.02	0.303*	0.008
Preoperative risk assessment (NSQIP)	0.241*	0.03	**0.204**	0.11
Frailty	0.09	0.54	0.085	0.55
METs	0.09	0.53	0.081	0.48
Cancer	0.26	0.04	0.234	0.09
Postoperative creatinine increase	0.13	0.42	0.118	0.1
Intraoperative (duration in min)				
Heart rate > 100	0.051	0.65	−0.026	0.89
Heart rate < 55	0.057	0.61	0.056	0.70
Heart rate > 87	0.131	0.40	0.179	0.39
Systolic blood pressure < 100	0.171	0.38	0.155	0.35
Pulse pressure > 62	0.182	0.32	0.317* **p= 0.007**	0.03
Pulse pressure < 53	0.011	0.71	− 0.042	0.80
Mean arterial pressure < 75	0.319*	0.03	0.318*	0.03
Mean arterial pressure < 55	0.209	0.11	0.144	0.53

Data shown are Kendall’s correlation coefficient for tied ranks (Tau-B)

*p value < 0.05, actual value given

The data were stratified according to age (years) < 70 (*n* = 22) and > 70 (*n* = 28). This stratification is outlined in Table 4. Patients > 70 years had a correlation between duration MAP < 75 mmHg and prolonged LOS (tau = 0.571, *p* = 0.004).

**Table 4:** Correlations between patient characteristics, intraoperative haemodynamic indices and Comprehensive Complication Index (CCI) and hospital length of stay (LOS), stratified according to age

	Comprehensive Complication Index		Length of hospital stay	
Age (year)	< 70 *n* = 22	> 70 *n* = 28	< 70 *N* = 22	> 70 *N* = 28
Length of stay in hospital (total)	0.282	1	1	0.341* ***p*= 0.03**
Preoperative risk assessment (NSQIP)	0.067	0.341* *p* = 0.03	0.129	0.335* ***p*= 0.03**
Intraoperative (duration in min)				
Heart rate > 100	− 0.08	0.335* *p* = 0.02	− 0.078	0.336
Heart rate < 55	− 0.048	0.336	− 0.013	0.076
Heart rate > 87	0.042	0.076	0.083	0.266
Systolic blood pressure < 100	0.005	0.266	0.231	0.293
Pulse pressure > 62	− 0.021	0.293	0.289	0.273
Pulse pressure < 53	− 0.026	0.273	− 0.012	0.098
Mean arterial pressure < 75	− 0.036	0.098	0.386* ***p*= 0.02**	0.571* ***p*= 0.004**
Mean arterial pressure < 55	− 0.013	0.571* *p* = 0.004	0.056	0.336*
PACU				
Heart rate > 100	− 0.096	0.336* *p* = 0.03	0.004	0.103
Heart rate < 55	− 0.219	0.027	− 0.055	− 0.222
Heart rate > 87	− 0.026	− 0.011	− 0.051	0.305
Systolic blood pressure < 100	0.065	0.146	0.143	0.27
Pulse pressure > 62	− 0.181	0.013	0.027	− 0.081
Pulse pressure < 53	0.041	− 0.076	− 0.027	0.253
Mean arterial pressure < 75	0.108	−0.094	0.155	0.048
Mean arterial pressure < 55	0.122	0.093	0.141	0.112

Data shown are Kendall’s correlation coefficient for tied ranks (Tau-B)

*p value < 0.05, actual value given

The data were then stratified according to ASA score, as shown in Table 5. Patients with ASA > 3 had a significant association between CCI and duration of hypotension, regardless of the definition of hypotension. Therefore, the SAP duration patients had < 100 mmHg (tau = 0.277, *p* = 0.04), the MAP duration < 75 mmHg (tau = 0.48, *p* = 0.01), and the MAP duration < 55 mmHg (tau = 0.308, *p* = 0.03), were all associated with worse complications as measured by CCI. Similarly, ASA > 3 patients had a significant association between LOS and duration of pulse pressure (PP) > 62 (tau = 0.312, *p* = 0.03) or MAP < 75 mmHg (tau = 0.358, *p* = 0.02).

**Table 5:** Correlations between patient characteristics and intraoperative haemodynamic parameters and CCI and LOS, stratified according to ASA grade

	CCI		LOS	
	ASA 1–2 *n* = 31	ASA > 3 *n* = 19	ASA 1–2 *n* = 31	ASA > 3 *n* = 19
	Tau	Tau	Tau	Tau
General				
Comprehensive Complication Index	1	1	0.21	0.45
Length of stay in hospital (total)	0.384	0.401* **p= 0.002**	1	1
Preoperational risk assessment (NSQIP)	− 0.159	0.328* **p= 0.02**	− 0.08	0.349* **p= 0.008**
Age	0.317	0.270* **p= 0.04**	0.529* **p= 0.01**	0.226
Intraoperative				
Heart Rate > 100 (duration)	− 0.243	0.219	− 0.016	− 0.022
Heart Rate < 55 (duration)	0.369	− 0.005	**0.376**	− 0.05
Heart Rate > 87 (duration)	− 0.055	0.199	0.044	0.239
Systolic Blood Pressure < 100 (duration)	− 0.1	0.277* **p= 0.04**	− 0.274	0.268* *p* =
Pulse Pressure > 62 (duration)	− 0.073	**0.259** **p= 0.04**	0.335	0.312*
Pulse Pressure < 53 (duration)	− 0.186	0.11	− 0.457* **p= 0.02**	0.069
Mean Arterial Pressure < 75 (duration)	− 0.057	0.480* **p= 0.01**	0.171	0.358* **p= 0.02**
Mean Arterial Pressure < 55 (duration)	− 0.05	0.308* **p= 0.03**	0.106	0.153
PACU				
Heart Rate > 100 (duration)	− 0.518* **p= 0.005**	− 0.019	− 0.482* **p= 0.01**	0.017
Heart Rate < 55 (duration)	0.074	0.09	0.177	− 0.025
Heart Rate > 87 (duration)	− **0.499** p= 0.01*	0.145	− 0.301	0.142
Systolic Blood Pressure < 100 (duration)	− 0.22	0.178	0.088	0.314* **p= 0.03**
Pulse Pressure > 62 (duration)	− 0.215	0.035	− 0.023	0.153
Pulse Pressure < 53 (duration)	− 0.29	− 0.059	− 0.104	0.083
Mean arterial pressure < 75 (duration)	− 0.144	0.246	0	0.259* **p= 0.04**
Mean arterial pressure < 55 (duration)	− 0.299	0.301* **p= 0.03**	− 0.042	0.326* **p= 0.03**

Data shown are Kendall’s correlation coefficient for tied ranks (Tau-B)

*p value < 0.05, actual value given

The stratification based on NSQIP scores is detailed in Table 6. Among higher risk patients (NSQIP > 15), CCI was correlated closely with longer duration of MAP < 55 mmHg (tau = 0.521, *p* = 0.005) and MAP < 75 mmHg (tau = 0.378, *p* = 0.009). LOS also correlated with duration with PP > 62 mmHg (tau = 0.380, *p* = 0.009) in this subset of patients.

**Table 6:** Correlations between patient characteristics and intraoperative haemodynamic parameters and CCI and LOS, stratified according to NSQIP (> 15)

	CCI		LOS	
	NSQIP < 15 *N* = 26	NSQIP > 15 *N* = 24	NSQIP < 15	NSQIP > 15
Comprehensive Complication Index	1	1	0.28	0.49
Age	**tau = 0.33,** **p= 0.04**	0.239	0.195	0.353* ***p*= 0.04**
Intraoperative				
Heart rate > 100 (duration)	− 0.186	0.16	0.129	− 0.234
Heart rate < 55 (duration)	0.364* ***p*= 0.04**	− 0.074	0.044	0.146
Heart rate > 87 (duration)	− 0.019	0.195	0.304	− 0.037
Systolic blood pressure < 100 (duration)	0.177	0.205	0.058	0.255
Pulse pressure > 62 (duration)	0.056	0.245	0.303	0.380* ***p*= 0.009**
Pulse pressure < 53 (duration)	0.101	− 0.032	− 0.152	0.019
Mean arterial pressure < 75 (duration)	0.202	0.378* ***p*= 0.03**	0.238	0.370* ***p*= 0.03**
Mean arterial pressure < 55 (duration)	− 0.113	0.521* ***p*= 0.005**	0.078	0.272
PACU^				
Heart rate > 100 (duration)	− 0.165	− 0.081	− 0.005	− 0.125
Heart rate < 55 (duration)	0.044	0.066	0.054	0.05
Heart rate > 87 (duration)	− 0.212	0.125	− 0.055	0.096
Systolic blood pressure < 100 (duration)	− 0.11	0.061	0.285	0.026
Pulse pressure > 62 (duration)	− 0.005	− 0.125	0.14	0.082
Pulse pressure < 53 (duration)	− 0.256	− 0.053	0.004	0.008
Mean arterial pressure < 75 (duration)	0.071	0.109	0.152	0.071
Mean arterial pressure < 55 (duration)	− 0.025	0.268	0.065	0.3

This is the risk (%) of perioperative cardiac events using the ACS-NSQIP prediction tool that was being used at the time of the MET-REPAIR study. Data shown are Kendall’s correlation coefficient for tied ranks (Tau-B)

*p value < 0.05, actual value given

^None of the Kendall’s correlation coefficient for tied rank (Tau-B) values in Post Anaesthesia Care Unit (PACU) reached statistical significance

There was no correlation between preoperative NT-proBNP and either CCI (Spearman’s correlation coefficient *r* = − 0.01, 95% CI − 0.30 to − 0.28, *p* = 0.93) or LOS (*r* = − 0.09, 95% CI − 0.02 to − 0.38, *p* = 0.54). Neither was there any association between preoperative or postoperative creatinine and either CCI or LOS.

## Discussion

This retrospective analysis has shown, for the first time to our knowledge, a negative correlation between duration of hypotension (variously defined as MAP < 75, MAP < 55 or SAP < 100 mmHg) and increased postoperative morbidity, expressed in terms of increased CCI or length of stay, among higher risk patients. There was also a positive correlation between duration of PP > 62 mmHg intra-operatively during anaesthesia and increased CCI. A PP > 62 mmHg may result from a high systolic arterial pressure or a low diastolic arterial pressure. While this is often attributed to age-related atherosclerosis, its significance in the intraoperative period is unclear, and the mechanism by which wide pulse pressure could cause organ impairment and hence postoperative morbidity is uncertain. Increased PP > 62 mmHg in the preoperative period has been shown to be an independent risk factor for overall postoperative morbidity and myocardial injury during noncardiac surgery in these increased risk patients, while SAP was not (Abbott et al. 2017a; Abbott et al. 2017b; Abbott et al. 2019a; Ackland et al. 2018). Elevated PP has been suggested to increase myocardial stress, promote left ventricular hypertrophy, impair coronary perfusion and increase aortic stiffness. Increased pulse pressure causes dynamic stretch on the vascular endothelium which in turns causes shear stress—a recognized factor in the pathogenesis of atherosclerosis and linked to coronary events (Safar et al. 2011).

Previous large-scale observational studies have shown an association between some of these haemodynamic parameters measured in the preoperative period and postoperative morbidity or mortality. The VISION study, a prospective, observational study of > 15,000 increased risk patients undergoing noncardiac surgery, identified an association between elevated troponin (> 30 pg ml^-1^) and myocardial injury after noncardiac surgery (MINS) (Botto et al. 2014).

In addition, dysfunction of the sympathetic nervous system at baseline before surgery has also been associated both with increased postoperative morbidity (Abbott et al. 2019a). This is distinct from parasympathetic vagal dysfunction—a secondary analysis of the VISION database has suggested a link between loss of vagal tone preoperatively and MINS (Abbott et al. 2019b; May et al. 2019). Impairment of heart rate recovery (HRR), which should be > 12 beats per minute from maximum heart rate achieved at maximal oxygen consumption during cardiopulmonary exercise testing, is associated with adverse postoperative outcome (Ackland et al. 2019). Further, some signals of intraoperative tachycardia or bradycardia have also been linked to increased risk of adverse events (Abbott et al. 2019b; Ackland et al. 2019; May et al. 2019; Wijeysundera et al. 2018).

The value of intraoperative haemodynamic parameters (during anaesthesia) in influencing postoperative outcomes became apparent when a prospective, randomised clinical trial of increased risk patients showed that maintaining intraoperative SAP > 90% of patients’ individual preoperative baseline reduced 30-day postoperative morbidity by approximately one-third (Futier et al. 2017). Our present study suggests that other haemodynamic parameters, especially the duration of absolute PP > 62 mmHg and MAP < 75 mmHg, may also be associated with increased morbidity. Intraoperative heart rate (HR) > 87 and HR > 100 have in previous publications cited, been independently linked with specific adverse postoperative morbidity, particularly MINS (Abbott et al. 2017a; Abbott et al. 2017b). Therefore, we felt it important to include both in our analysis of overall morbidity.

The recent Perioperative Quality Initiative on Intraoperative Blood Pressure has recommended maintaining SAP > 75 mmHg to minimise MINS and all postoperative morbidity including acute kidney injury and yet no definitive conclusions on a cut off MAP value can be given (Sessler et al. 2019). Even short durations of hypotension may be linked with adverse postoperative outcomes. However, defining the threshold of hypotension is unclear with MAP < 80 mmHg, < 70 mmHg and < 65 mmHg, even for durations of < 10 min, being implicated in the development of postoperative complications up to 30 days (Kristensen et al. 2014a; Kristensen et al. 2014b; Sessler et al. 2019; Wesselink et al. 2018). Whether large database studies of intraoperative haemodynamic parameters will enable prediction of postoperative morbidity remains to be determined.

A large observational study of > 60,000 hip fracture patients has highlighted the value of observing the association between intra-operative MAP thresholds and 30-day mortality and morbidity. However, it was noted that 30-day mortality may be a rather insensitive end-point of postoperative complications compared with postoperative morbidity indices (White et al. 2014). Short durations of low intra-operative MAP are associated with AKI and myocardial injury in large observational studies (Walsh et al. 2013). Our own data did not reflect this, perhaps because of reduced power from the relatively low number of patients included. Further, we found no association between the preoperative serum biomarker of adverse cardiac outcome, NT-proBNP, and postoperative morbidity. This may reflect the fact that CCI measures overall all-cause morbidity, whereas NT-proBNP is predictive of cardiac morbidity (Choi et al. 2010). All values observed in our present study were low and no major adverse cardiac event was observed in this study. Neither was there any association between preoperative or postoperative creatinine and either CCI or LOS.

We recognise the limitations of our small dataset and acknowledge that our univariate analysis of factors affecting the Comprehensive Complications Index may yield spurious results, which is why we emphasised the results stratified for age and risk. In addition, the retrospective design limits the extent to which our findings can be interpreted and generalised. Length of stay as an outcome is being replaced by the more patient-centred outcomes such as DAOH30, which would also have been a suitable end-point for this study (Moonesinghe et al. 2019).

Indeed this study shows only an association between intraoperative hypotension and increased postoperative morbidity. Demonstrating a causal relationship would require a prospective, randomised controlled trial to test the hypothesis that treating intraoperative hypotension immediately at the thresholds indicated in our work reduces postoperative morbidity. We believe this is warranted. Nonetheless, investigating clinical research questions such as this increases the input of anaesthesiologists into perioperative medicine, which has the potential to improve postoperative outcomes for patients (Bollen Pinto et al. 2019).

In conclusion, our findings suggest a signal that intraoperative PP > 62 mmHg and MAP < 75 mmHg in particular, are associated with increased risk of postoperative morbidity. These findings should be reevaluated in a large-scale database. If confirmed, trials to investigate whether intraoperative interventions to normalise the haemodynamic parameters (such vasodilator or vasoconstrictor therapy) can reduce postoperative morbidity would be warranted.

## Data Availability

The datasets used and/or analysed during the current study are available in our institution and can be requested from the corresponding author on reasonable request.
