# Correction to: Can routine perioperative haemodynamic parameters predict postoperative morbidity after major surgery?

**DOI:** 10.1186/s13741-020-00149-1

**Published:** 2020-05-29

**Authors:** Jean-Francois Bonnet, Eleanor Buggy, Barbara Cusack, Aislinn Sherwin, Tom Wall, Maria Fitzgibbon, Donal J. Buggy

**Affiliations:** 1 Department of Anaesthesiology & Perioperative Medicine, Mater University Hospital, School of Medicine, University College Dublin, Dublin, Ireland; 2 Department of Medical Biochemistry, Mater University Hospital, School of Medicine, University College Dublin, Dublin, Ireland

**Correction to: Perioper Med (2020) 9: 9**

**https://doi.org/10.1186/s13741-020-0139-6**

Following publication of the original article (Bonnet et al. 2020), the authors identified an error in the methods section.

In the Methods section, we stated that the patients studied in our single centre were already in the ongoing MET-Repair study: In fact, the patient cohort we described was a separate local group of patients with similar enrollment criteria to MET-Repair, but our outcome measures were entirely different as reported in our study.
